# Trends of serum 25(OH) vitamin D and association with cardiovascular disease and all-cause mortality: from NHANES survey cycles 2001–2018

**DOI:** 10.3389/fnut.2024.1328136

**Published:** 2024-02-02

**Authors:** Changlun Hu, Mei Yang

**Affiliations:** Department of Internal Medicine, Chongqing Nanan District Traditional Chinese and Western Medicine Hospital, Chongqing, China

**Keywords:** serum vitamin D concentration, all-cause mortality, CVD mortality, NHANES, trends

## Abstract

**Background:**

The focus of this survey is on survey data for adults aged 20 and above, covering nine survey cycles from 2001 to 2018. Additionally, the present study explored the correlation between vitamin D concentrations and both cardiovascular disease (CVD) and all-cause mortality.

**Objective:**

The objectives of this study were to evaluate the trend of changes in the serum 25(OH)D concentration changes in US adults during the survey period, the prevalence of vitamin D deficiency, and the current status of vitamin D dietary intake and supplementation.

**Methods:**

In-home health interviews were performed using meticulously designed questionnaires that gathered information on demographic details, socioeconomic conditions, dietary patterns, and overall health status. Health assessments were conducted in specially designed mobile centers.^1^

**Results:**

Survey data from 2001 to 2018 revealed a rise in serum 25(OH)D levels, from a weighted mean (95% CI) of 65.6 (63.8–67.4) nmol/L during 2001–2002 to 73.5 (70.4–76.5) nmol/L during 2017–2018, among US adults, while overall vitamin D deficiency rates remained stable (*p* = 0.152). Notably, in adults aged 20–39, 25(OH)D levels decreased (*p* = 0.002 for trend), and 25(OH)D deficiency increased (*p* = 0.003 for trend), especially among those with low incomes (deficiency >30%). Upon multivariable adjustment, an L-shaped relationship was found between serum 25(OH)D concentrations and both CVD and all-cause mortality (*p* < 0.001 for nonlinearity), as corroborated by sensitivity analyses.

**Conclusion:**

From 2001 to 2018, US adults experienced a significant increase in their serum 25(OH) D concentration. However, subgroups of individuals, including young adults and individuals with lower socioeconomic status, exhibited a heightened risk of 25(OH)D deficiency. Furthermore, an L-shaped relationship was found between 25(OH)D concentration and both all-cause and CVD mortality among US adults.

## 1 Introduction

Vitamin D, a lipophilic nutrient, is primarily obtained from dietary intake and is synthesized in skin tissue. This nutrient plays a crucial role in several vital physiological processes, including calcium and phosphate homeostasis, bone metabolism, immune modulation, and diverse cellular activities (1, 2). Deficits in the serum 25(OH)D concentration have been associated with osteoporosis, metabolic syndrome, cardiovascular disease, chronic kidney disease, asthma, and respiratory tract infections (3–5). Hence, exploring the impacts of serum 25(OH)D concentration, vitamin D ingestion, and supplementation on public health is a significant scientific endeavor.

An investigation from 1988 to 2010 demonstrated an increasing trend in the serum 25(OH)D concentration over time, with disparities among races, sexes, and age groups (6). Another study investigated the trends in vitamin D deficiency from 2001 to 2018 using NHANES data. However, this study did not explore the trends in vitamin D concentrations or deficiency rates over time across different age groups, educational levels, or income strata. Understanding these trends is essential for obtaining comprehensive insight into the public’s nutritional status (7). In addition, a large number of randomized controlled trials (RCTs) and meta-analyses have reported the impact of vitamin D supplementation on disease progression and specific mortality (8–11). However, the existing evidence from RCTs and meta-analyses only supports the survival benefits of vitamin D supplementation in targeted populations, such as elderly COVID-19 patients or cancer sufferers (12, 13), but fails to reveal benefits for unscreened populations in clinical studies (11). In fact, due to ethical considerations, existing clinical evidence cannot be used to determine whether vitamin D supplementation can benefit patients with vitamin D deficiency. Therefore, utilizing cycle-based nutritional survey data to investigate the relationship between the serum vitamin D concentration and mortality can serve as an important complement to RCT research.

The National Health and Nutrition Examination Survey (NHANES) is a key resource for public health research and has provided health and nutritional data for the US population since 1960 (14). This database provides valuable research data, such as serum 25(OH)D concentration, dietary and supplementary vitamin D intake, and related mortality data. Our study focused mainly on the trend of changes in the serum 25(OH)D concentration (25(OH)D2 + 25(OH)D3) in the US population, the prevalence of 25 (OHD) deficiency throughout the NHANES survey cycle, and the trend of vitamin D intake through diet and supplementation. Additionally, we also investigated the associations between serum 25(OH)D levels and cardiovascular disease (CVD) and all-cause mortality. Its purpose is to provide guidance for population health management.

## 2 Methods

### 2.1 Study design and population

The NHANES study protocol was approved by the Research Ethics Review Board of the National Center for Health Statistics (NCHS) of the Centers for Disease Control and Prevention. In-home health interviews were performed using meticulously designed questionnaires that gathered information on demographic details, socioeconomic conditions, dietary patterns, and overall health status. Health assessments were conducted in specially designed mobile centers. These evaluations included comprehensive evaluations of medical, dental, and physiological parameters and were, supplemented by laboratory tests performed by a professional medical team (14). We used open access data from the NHANES database. There was no financial incentive or reward for participation in the NHANES project. Informed consent form was signed by all participants at the time of their recruitment. In alignment with National Institutes of Health (NIH) guidelines, our study did not involve direct interaction with the participants and hence was not categorized as involving human subjects. The focus of this survey is on survey data for adults aged 20 and above, covering nine survey cycles from 2001 to 2018. The objectives of this study included evaluating the trend of changes in the serum 25(OH)D concentration changes in US adults during the survey period, the prevalence of vitamin D deficiency, and the current status of vitamin D dietary intake and supplementation. Additionally, present the study examined the correlation between vitamin D concentrations and both CVD and all-cause mortality.

### 2.2 Evaluation of serum 25(OH)D levels and definition of 25(OH)D deficiency

The methods used to determine the serum 25 (OH) D concentrations and regression used equations are detailed in https://wwwn.cdc.gov/nchs/nhanes/vitamind/analyticalnote.aspx. Based on previous reports, this study defined vitamin D deficiency as a serum 25 (OH) D level less than 50 nmol/L (15).

### 2.3 Assessment of vitamin D intake

To gather dietary information, participants engaged in 24-h dietary recall interviews. The initial interview was performed at the Mobile Examination Center (MEC), followed by a telephone interview 3 to 10 days later. This process provides a reliable means to survey the dietary habits of the study population. Dietary and supplementary intake data on vitamin D from individuals were collected on the first day of the interviews.

### 2.4 Extraction of CVD and all-cause mortality

We combined NHANES data with data from the US National Death Index up to 2019 to determine the trend of mortality rates over time. All-cause mortality included all potential causes of death but was not limited to the analysis of specific causes. The definition of CVD mortality was defined according to the tenth revision of the International Classification of Diseases and Related Health Problems (encapsulating codes I00 to I09, I11, I13, I20 to I51, and I60 to I69) (16).

### 2.5 Assessment of covariates

Standard questionnaires were used to obtain covariates such as age, sex, race/ethnicity, education attainment, poverty income ratio (PIR), smoking status, physical activity, and health status. Anthropometric data such as body weight and height, as well as alcohol consumption data, were obtained from mobile centers (17).

To evaluate alcohol consumption, individuals were divided into two groups: the nonalcoholic group (drinking <12 alcohol drinks for 1 year) and the alcoholic group. By answering “Have you smoked at least 100 cigarettes in your lifetime?” and “Do you smoke now?,” the study population was divided into nonsmokers, former smokers, or current smokers. The incidence rate of diseases, including malignant tumors, diabetes, hypertension and hypercholesterolemia. By integrating these parameters, the scope and accuracy of epidemiological investigations have been clarified.

### 2.6 Statistical analysis

This study complies with the Preferred Reporting Items for Complex Sample Survey Analysis (PRICSSA) guidelines. We used sample weighting, stratification and cluster analysis to ensure the representativeness of the study population and analyzed the trend of the serum 25(OH)D concentration as a continuous variable over a two-year period. The weight variable design, PSU/stratum and survey’s response rate are detailed in the NHANES.^2^ The serum 25(OH)D concentration and deficiency were measured with “WTMEC2YR” as the weighting variable, vitamin D intake from food and supplements was measured with “WTDRD1,” and mortality was measured with “WTMEC2YR” as the weighting variable. The unweighted sample size was reported. We utilized weighted linear regression analyses to determine the trends in the serum 25(OH)D concentration, as well as the trends in the intake of vitamin D from food and supplements. Weighted logistic regression analysis was used to estimate the trend of occurrence of serum 25(OH)D deficiency in each cycle. We employed Taylor series linearization for standard error calculations, and weighted averages and 95% confidence intervals were estimated for each follow-up cycle. We calculated the difference in estimated mean values between the first and last cycles and evaluated the trend of changes in the serum vitamin D concentration according to age, sex, PIR, education level, and ethnicity. We removed samples with missing values and reported the removal ratio. We deleted the group that retained only one primary sampling unit and reported the sample proportion of the deleted group. A weighted Cox multivariable regression model was used to calculate hazard ratios (HRs) and 95% confidence intervals (CIs) between serum 25(OH)D levels and CVD and all-cause mortality. The proportional hazards assumption was verified using Schoenfeld residual analysis. The correlation between serum 25(OH)D levels and all-cause mortality was explored using restricted cubic spline analysis, with likelihood ratio tests employed for nonlinear investigations. In this study, we developed three models: Model 1, a correlation between the serum 25(OH)D concentration and all-cause mortality without adjusting for covariates; Model 2, an adaptation of Model 1 with the inclusion of significant demographic and socioeconomic covariates; and Model 3, an extension of Model 2 with the addition of pertinent medical and lifestyle covariates. In the mortality sensitivity analysis, age -related structural variances were assessed using the standard set by the 2000 US Census population, and participants with less than 2 years of follow-up were omitted to mitigate reverse causality. For mortality correlation analysis, we stratified the serum vitamin D concentrations into four quartiles. The data were analyzed using R software (Version: 4.2.0), for which a *p*-value of <0.05 indicated statistical significance. We stratified the data by sex and separately investigated the relationship between serum 25(OH)D levels and mortality rates in male and female.

## 3 Results

### 3.1 Population characteristics

During the period from 2001 to 2002 to 2017 to 2018, serum 25(OH)D concentrations were recorded for 92.7% (44,461 out of 47,954) of the adult participants aged 20 and above. The selection process for the data can be found in Supplementary material S1. The proportion of individuals aged 60 and above in this study increased from 21.6 to 31.4%. In terms of education, the number of participants with education below high school declined from 19.5 to 11.3%, while the number of participants with education at university or above rose from 55.2 to 61.5%. In terms of ethnic group, the proportion of non-Hispanic white participants decreased from 71.4 to 62.2% (Table 1).

**Table 1:** Sociodemographic characteristics of US adults, 2001 to 2018.

	Percentage (%) of adults by year (95% CI)^a,b^								
Characteristics	2001–2002 (*n* = 5,027)	2003–2004 (*n* = 4,742)	2005–2006 (*n* = 4,773)	2007–2008 (*n* = 5,707)	2009–2010 (*n* = 6,059)	2011–2012 (*n* = 5,319)	2013–2014 (*n* = 5,588)	2015–2016 (*n* = 5,475)	2017–2018 (*n* = 5,265)
Age group, y									
20–39	38.6 (35.6–41.6)	36.7 (33.8–39.5)	36.3 (33.7–38.9)	35.4 (32.9–37.9)	34.6 (32.2–36.9)	34.5 (30.0–39.1)	34.5 (32.2–36.9)	35.0 (32.4–37.6)	34.4 (31.9–36.9)
40–59	39.8 (37.8–41.8)	39.5 (37.2–41.8)	39.2 (36.2–42.1)	39.5 (37.4–41.6)	39.1 (37.9–40.3)	37.5 (35.1–39.9)	36.5 (34.5–38.5)	35.1 (33.0–37.3)	34.2 (31.4–36.9)
≥60	21.6 (19.7–23.6)	23.9 (21.8–26.0)	24.6 (20.7–28.4)	25.1 (23.1–27.2)	26.3 (24.1–28.6)	28.0 (25.1–30.9)	28.9 (27.1–30.8)	29.9 (27.0–32.8)	31.4 (28.2–34.7)
Sex									
Male	47.8 (46.9–48.7)	47.9 (46.6–49.3)	48.1 (47.0–49.2)	48.2 (47.1–49.3)	48.2 (47.3–49.2)	48.0 (46.4–49.6)	48.1 (46.7–49.4)	48.1 (46.9–49.2)	48.1 (46.5–49.8)
Female	52.2 (51.3–53.1)	52.1 (50.7–53.4)	51.9 (50.8–53.0)	51.8 (50.7–52.9)	51.8 (50.8–52.7)	52.0 (50.4–53.6)	51.9 (50.6–53.3)	51.9 (50.8–53.1)	51.9 (50.2–53.5)
Income-to-poverty ratio^c^									
≤1	14.3 (12.5–16.0)	13.1 (10.6–15.6)	11.2 (9.7–12.7)	14.2 (12.0–16.5)	14.8 (13.0–16.7)	18.0 (14.6–21.4)	16.2 (12.8–19.6)	14.5 (11.8–17.2)	12.9 (10.8–15.1)
1–3.9	47.2 (44.4–50.0)	50.5 (47.4–53.5)	51.6 (48.5–54.6)	47.1 (43.9–50.3)	48.4 (45.5–51.4)	45.6 (41.0–50.3)	48.5 (45.5–51.5)	48.0 (44.0–52.0)	47.8 (43.6–52.0)
≥4	38.6 (34.8–42.3)	36.4 (32.3–40.6)	37.2 (33.4–41.1)	38.7 (34.2–43.2)	36.7 (34.4–39.1)	36.4 (30.6–42.1)	35.3 (30.2–40.3)	37.5 (31.6–43.4)	39.3 (35.1–43.4)
Education level^d^									
Less than high school	19.5 (17.5–21.5)	18.5 (16.3–20.7)	17.8 (15.0–20.5)	20.5 (17.6–20.4)	19.0 (17.1–21)	16.7 (13.4–20.1)	15.3 (12.2–18.4)	14.6 (11.2–17.9)	11.3 (9.7–12.9)
High school or equivalent	25.2 (23.5–26.9)	27.1 (25.1–29.0)	25.0 (23.5–26.6)	25.5 (23.0–28.0)	22.8 (20.6–25.1)	20.2 (17.6–22.8)	21.8 (19.4–24.2)	20.7 (18.5–22.8)	27.2 (24.1–30.2)
College or more	55.2 (52.1–58.3)	54.5 (51.9–57.0)	57.2 (53.5–60.9)	54.0 (49.3–58.8)	58.1 (55.3–61.0)	63.1 (58.1–68.1)	62.9 (58.8–67.0)	64.8 (60.2–69.3)	61.5 (57.6–65.4)
Race									
Mexican American	7.2 (5.5–8.9)	7.8 (4.0–11.6)	8.0 (6.0–9.9)	8.4 (5.5–11.2)	8.6 (4.3–12.9)	7.7 (4.4–11.1)	9.2 (5.7–12.6)	8.9 (4.7–13.0)	8.8 (5.6–12.0)
Non-Hispanic White	71.4 (66.5–76.3)	72.1 (65.4–78.7)	71.8 (66.4–77.3)	69.4 (62.3–76.6)	67.9 (61.4–74.5)	66.4 (58.8–74.0)	65.8 (59.4–72.2)	63.8 (56.3–71.4)	62.2 (57.2–67.3)
Non-Hispanic Black	10.9 (7.5–14.3)	11.2 (7.6–14.8)	11.5 (7.7–15.3)	11.3 (7.6–15.0)	11.4 (9.7–13.1)	11.5 (7.1–15.9)	11.4 (8.3–14.6)	11.4 (7.1–15.6)	11.4 (8.3–14.6)
Other	10.5 (6.1–14.8)	9.0 (6.9–11.0)	8.7 (6.5–10.9)	10.9 (7.5–14.4)	12.1 (8.6–15.6)	14.3 (11.3–17.4)	13.6 (11.2–16.0)	15.9 (12.5–19.3)	17.5 (14.3–20.7)

^a^Percentages were adjusted for NHANES survey weights.^b^The proportional sum may not be equal to 1 in some cycles due to rounding.^c^4,115 (8.6%) samples reported income-to-poverty ratio (PIR) missing value.^d^68 (0.1%) samples reported education level missing value.

### 3.2 Trends in the serum 25(OH)D concentration

In the current study population, we found a significant increase in the serum 25(OH)D concentration, from 65.6 nmol/L (95% CI, 63.8–67.4 nmol/L) in the 2001–2002 cycle to 73.5 nmol/L (95% CI, 70.4–76.5 nmol/L) in the 2017–2018 cycle (*p* < 0.001 for trend; Table 2). Although the serum 25(OH)D concentration showed an increasing trend, the incidence rate of 25(OH)D deficiency remained relatively stable [22.7% (95% CI, 19.7–25.8%) for 2001–2002 and 21.7% (95% CI, 18.1–25.4%) for 2017–2018] (*p* = 0.152 for trend; Table 3). According to the subgroup analysis of sex and age, the serum 25(OH)D concentration in the ≥60-year-old (*p* < 0.001 for trend) and female (*p* < 0.001 for trend) populations also showed an upward trend. Conversely, in the cohort aged 20–39 years, the exhibited a decreasing serum 25(OH)D concentration decreased trajectory from 66.2 nmol/L (95% CI, 64.3–68.1 nmol/L) in 2001–2002 to 61.9 nmol/L (95% CI, 59.1–64.8 nmol/L) in 2017–2018 (*p* < 0.002 for trend; Table 2). During the survey period, there was no significant change in the trend of serum 25(OH)D concentration in individuals with a PIR ≤ 1 (*p* = 0.325 for trend; Table 2), while the incidence of 25(OH)D deficiency remained above 30% (Table 3). A significant increase trend in the 25(OH)D concentration was noted among individuals with PIRs 1–3.9 and ≥ 4 (*p* = 0.002 and < 0.001 for trend, respectively, Table 2). The PIR ≥4 subgroup exhibited a decrease in the occurrence of serum 25(OH)D deficiency, from 15.8% (95% CI, 12.1–19.6%) in 2001–2002 to 13.4% (95% CI, 10.0–16.8%) in 2017–2018 (Table 3), suggesting at a notable association between the incidence of 25(OH)D deficiency and poverty level. According to the stratified analysis of population education level, there was no significant change in the trend of serum 25(OH)D concentration or the incidence of vitamin D deficiency among individuals with lower levels of university education.

**Table 2:** Trends of serum vitamin D concentration (nmol/L) among adults, 2001 to 2018.

	Trends of serum vitamin D concentration (nmol/L), weighted mean (95%CI)^a^									Difference, 2017–2018 vs. 2001–2002 (95% CI)^b^	*P*-value for trend
	2001–2002 (*n* = 5,027)	2003–2004 (*n* = 4,742)	2005–2006 (*n* = 4,773)	2007–2008 (*n* = 5,707)	2009–2010 (*n* = 6,059)	2011–2012 (*n* = 5,319)	2013–2014 (*n* = 5,588)	2015–2016 (*n* = 5,475)	2017–2018 (*n* = 5,265)		
Overall	65.6 (63.8–67.4)	63.2 (60.0–66.4)	67.4 (65.4–69.5)	67.1 (65.2–69.1)	67.7 (65.0–70.4)	70.8 (67.7–73.9)	69.6 (67.0–72.1)	72.0 (68.8–75.3)	73.5 (70.4–76.5)	7.88 (4.19–11.6)	<0.001
Age											
20–39	66.2 (64.3–68.1)	63.6 (60.0–67.1)	69.0 (66.3–71.8)	66.0 (62.7–69.3)	63.4 (62.3–66.5)	62.9 (59.8–66.0)	61.6 (58.6–64.6)	62.6 (59.3–66.0)	61.9 (59.1–64.8)	−4.24 (−7.78--0.70)	0.002
40–59	65.8 (63.7–68.0)	62.8 (59.0–66.7)	66.7 (64.5–68.9)	67.0 (64.2–69.6)	68.7 (66.1–71.3)	70.9 (66.6–75.2)	68.9 (66.0–71.7)	71.7 (68.2–75.2)	72.8 (69.6–76.0)	6.99 (3.00–11.0)	<0.001
≥60	64.0 (61.8–66.1)	63.2 (61.0–65.4)	66.0 (63.9–68.2)	69.0 (66.9–71.1)	72.6 (69.5–75.7)	81.7 (78.3–85.1)	81.4 (78.7–84.1)	84.3 (80.9–87.8)	88.3 (85.2–91.5)	24.4 (20.4–28.3)	<0.001
Sex											
Male	66.3 (64.5–68.2)	63.6 (60.2–67.0)	67.3 (65.5–69.1)	65.5 (63.1–67.9)	65.6 (63.0–68.2)	67.7 (64.5–70.9)	65.8 (63.6–68.1)	68.2 (65.5–70.9)	70.2 (67.4–73.0)	3.86 (0.40–7.32)	0.09
Female	64.9 (62.8–67.0)	62.9 (59.8–65.9)	67.6 (65.0–70.1)	68.7 (66.8–70.6)	69.6 (66.6–72.7)	73.7 (70.2–77.2)	73.1 (69.8–76.3)	75.6 (71.4–79.8)	76.5 (73.0–80.0)	11.6 (7.31–15.9)	<0.001
Income-to-poverty ratio											
≤1	60.5 (57.6–63.4)	57.0 (52.2–61.8)	61.6 (57.9–65.3)	61.2 (57.4–65.1)	60.0 (57.4–62.5)	62.2 (60.0–64.5)	59.8 (56.6–63.1)	60.9 (56.6–65.2)	64.2 (61.1–67.4)	3.8 (−0.69–8.20)	0.325
1–3.9	64.3 (62.6–66.0)	62.2 (59.2–65.3)	66.5 (64.4–68.7)	66.5 (62.9–68.1)	65.9 (62.3–69.5)	68.9 (65.3–72.5)	67.7 (65.2–70.2)	69.2 (66.3–72.2)	70.8 (66.7–74.8)	6.48 (1.87–11.09)	0.002
≥4	69.9 (67.4–72.4)	67.1 (63.4–70.9)	70.5 (68.1–72.9)	71.5 (69.9–73.2)	73.8 (71.4–76.2)	77.3 (74.0–80.6)	76.7 (73.3–80.0)	79.4 (75.3–83.5)	80.7 (77.0–84.4)	10.8 (6.2–15.4)	<0.001
Education level											
Less than high school	60.9 (58.6–63.1)	57.3 (52.9–61.7)	61.9 (58.9–64.9)	62.1 (57.6–66.5)	61.9 (59.0–64.8)	66.1 (60.1–72.0)	64.3 (61.2–67.3)	63.9 (60.0–67.8)	65.3 (60.8–69.9)	4.45 (−0.85–9.76)	0.147
High school or equivalent	65.9 (64.1–67.7)	64.6 (61.2–68.1)	66.5 (64.1–68.9)	67.8 (65.2–70.4)	67.3 (64.4–70.1)	68.2 (64.8–71.7)	68.2 (64.4–72.0)	69.4 (65.4–73.3)	71.2 (67.2–75.1)	5.29 (0.74–9.84)	0.241
College or more	67.1 (64.9–69.3)	64.5 (61.1–67.8)	69.5 (67.5–71.5)	68.7 (66.6–70.9)	69.8 (67.1–72.6)	72.8 (69.7–75.9)	71.3 (69.0–73.7)	74.6 (71.4–77.9)	76.0 (73.1–78.8)	8.90 (5.13–12.7)	<0.001
Race^c^											
Mexican American^d^	56.9 (55.0–58.7)	54.5 (51.2–57.9)	58.3 (54.6–61.9)	54.1 (50.4–57.8)	54.2 (52.8–55.7)	54.4 (50.2–58.5)	55.4 (51.2–59.6)	55.2 (52.8–57.6)	57.3 (54.5–60.1)	0.42 (−3.15–4.00)	0.276
Non-Hispanic White^e^	70.1 (68.1–72.1)	68.5 (65.3–71.7)	72.1 (70.2–73.9)	73.9 (72.1–72.6)	74.8 (72.4–77.2)	78.0 (75.3–80.6)	76.0 (73.4–78.6)	79.9 (77.5–82.4)	81.0 (77.6–84.4)	10.9 (6.79–15.0)	<0.001
Non-Hispanic Black^f^	43.6 (42.5–44.7)	41.6 (38.8–44.3)	49.0 (46.9–51.0)	42.0 (39.2–44.9)	46.5 (42.0–51.0)	50.8 (48.4–53.1)	50.4 (47.5–53.2)	51.6 (49.3–53.8)	54.7 (51.7–57.8)	11.1 (7.74–14.6)	<0.001
Other^g^	58.6 (55.4–61.9)	53.4 (50.7–56.2)	60.3 (57.2–63.4)	56.8 (53.9–59.8)	55.6 (52.8–58.5)	61.1 (58.4–63.8)	63.1 (61.4–64.8)	63.4 (60.2–66.6)	66.6 (63.7–69.5)	7.93 (3.39–12.5)	<0.001

^a^Data were adjusted for NHANES survey weights to be nationally representative.^b^Values may not equal the difference between the beginning and ending estimates because of rounding.^c^Stratification by ethnic characteristics leaded to stratum with a single PSU. Samples in the stratum with the singleton PSU were removed.^d^261 (3.3%) samples removed.^e^38 (0.2%) samples removed.^f^147 (1.4%) samples removed.^g^68 (0.8%) samples removed.

**Table 3:** Trends in the weighted percentage of individuals with vitamin D deficiency among adults, 2001 to 2018.

	Weighted percentage (%) of individuals with vitamin D deficiency, % (95% CI)^a^									*P*-value for trend
	2001–2002 (*n* = 5,027)	2003–2004 (*n* = 4,742)	2005–2006 (*n* = 4,773)	2007–2008 (*n* = 5,707)	2009–2010 (*n* = 6,059)	2011–2012 (*n* = 5,319)	2013–2014 (*n* = 5,588)	2015–2016 (*n* = 5,475)	2017–2018 (*n* = 5,265)	
Overall	22.7 (19.7–25.8)	30.5 (24.5–36.5)	21.5 (17.8–25.1)	26.1 (22.4–29.7)	25.7 (21.9–29.5)	24.5 (19.5–29.6)	24.5 (20.8–28.2)	23.1 (18.8–27.4)	21.7 (18.1–25.4)	0.152
Age										
20–39	22.4 (18.8–26.1)	32.6 (25.7–39.5)	20.6 (16.1–25.2)	29.4 (23.6–35.2)	30.8 (25.6–36.0)	32.3 (25.9–38.7)	32.1 (26.9–37.2)	31.7 (25.7–37.7)	31.6 (27.0–36.2)	0.003
40–59	22.4 (18.8–25.9)	29.6 (22.6–36.6)	22.6 (17.8–27.5)	25.0 (21.0–29.0)	24.1 (19.9–28.2)	23.7 (17.8–29.6)	24.3 (19.7–28.8)	21.6 (17.0–26.2)	19.8 (15.7–23.9)	0.076
≥60	24.1 (20.2–27.9)	28.5 (24.7–32.3)	20.8 (17.3–24.3)	22.9 (19.2–26.6)	20.7 (18.0–23.4)	14.8 (11.2–18.5)	14.7 (12.3–17.0)	14.0 (11.3–16.7)	12.0 (9.1–14.9)	<0.001
Sex										
Male	19.6 (17.1–22.2)	26.9 (20.5–33.3)	19.6 (16.2–23.0)	24.3 (20.1–28.6)	24.3 (20.1–28.5)	24.7 (18.8–30.7)	25.8 (21.5–30.1)	23.5 (19.3–27.8)	23.1 (18.6–27.6)	0.364
Female	25.7 (21.7–29.6)	33.9 (27.9–39.8)	23.2 (19.0–27.5)	27.7 (24.1–31.3)	27.0 (23.1–31.0)	24.3 (19.6–29.0)	23.3 (19.5–27.1)	22.6 (17.7–27.5)	20.5 (17.2–23.8)	<0.001
Income-to-poverty ratio										
≤1	33.6 (26.9–40.3)	41.6 (31.0–52.2)	31.8 (25.3–38.4)	37.7 (29.8–45.6)	36.7 (31.5–41.9)	33.9 (28.0–39.8)	38.3 (32.4–44.1)	37.1 (30.0–44.1)	33.9 (28.7–39.0)	0.896
1–3.9	24.6 (21.5–27.7)	32.9 (26.4–39.4)	22.7 (19.0–26.4)	27.9 (23.7–32.1)	28.5 (23.5–33.5)	27.1 (20.8–33.5)	26.5 (22.5–30.4)	25.5 (20.4–30.6)	23.6 (18.5–28.6)	0.2667
≥4	15.8 (12.1–19.6)	22.6 (16.5–28.7)	15.6 (11.4–19.7)	19.0 (15.9–22.1)	16.4 (12.6–20.2)	16.5 (12.6–20.4)	15.3 (11.6–19.0)	14.6 (10.4–18.8)	13.4 (10.0–16.8)	0.024
Education level										
Less than high school	32.2 (28.2–36.1)	40.5 (30.5–50.6)	31.7 (26.0–37.4)	33.9 (26.4–41.3)	33.4 (28.8–38.0)	29.8 (20.9–38.8)	32.1 (26.3–37.9)	31.8 (25.6–37.9)	30.7 (25.5–35.9)	0.144
High school or equivalent	23.4 (19.6–27.1)	27.4 (21.1–33.7)	23.3 (18.7–28.0)	25.2 (20.7–29.7)	25.9 (22.3–29.6)	29.6 (22.5–36.6)	26.2 (21.4–31.1)	24.9 (19.1–30.8)	23.2 (19.2–27.3)	0.953
College or more	19.2 (15.7–22.7)	28.7 (22.7–34.8)	17.5 (13.9–21.2)	23.6 (19.7–27.5)	23.0 (18.8–27.2)	21.6 (17.2–25.9)	22.0 (18.6–25.5)	20.6 (16.7–24.5)	19.4 (15.7–23.2)	0.279
Race^b^										
Mexican American^c^	33.8 (28.9–38.7)	43.5 (35.9–51.2)	34.6 (26.2–42.9)	43.7 (34.3–53.0)	43.1 (38.8–47.4)	44.0 (32.6–55.4)	41.1 (31.4–50.8)	45.2 (39.2–51.3)	40.2 (34.5–46.0)	0.174
Non-Hispanic White^d^	14.0 (11.5–16.4)	21.1 (16.1–26.1)	12.7 (9.7–15.7)	15.9 (13.8–18.0)	14.7 (11.9–17.4)	14.3 (10.6–18.0)	15.5 (12.0–18.9)	12.4 (9.7–15.1)	12.2 (8.7–15.7)	0.027
Non-Hispanic Black^e^	72.4 (69.5–75.3)	71.5 (64.5–78.5)	60.8 (54.1–67.5)	70.7 (64.8–76.6)	64.4 (55.3–73.4)	58.3 (54.2–62.3)	58.3 (53.2–63.5)	56.3 (51.6–61.1)	53.1 (46.7–59.5)	<0.001
Other^f^	32.6 (25.3–40.0)	46.7 (39.1–54.4)	32.7 (26.1–39.2)	38.0 (29.6–46.4)	42.0 (35.3–48.5)	36.5 (30.4–42.6)	30.4 (25.9–35.0)	31.1 (26.6–35.6)	26.9 (23.2–30.6)	<0.001

^a^Data were adjusted for NHANES survey weights to be nationally representative.^b^Stratification by ethnic characteristics leaded to stratum with a single PSU. Samples in the stratum with the singleton PSU were removed.^c^261 (3.3%) samples removed.^d^38 (0.2%) samples removed.^e^147 (1.4%) samples removed.^f^68 (0.8%) samples removed.

### 3.3 Trends in dietary supplements and vitamin D

During the survey period from 2007 to 2018, the intake of vitamin D in dietary supplements increased (Supplementary material S2). Regarding the survey of food vitamin D intake, we found that during the 2017–2018 period, the total study population had lower vitamin D intake (difference: −0.13 nmol/L; 95% CI, −0.52 to 0.26 nmol/L). However, vitamin D intake was greater in the other survey cycles than in the compared to 2007–2008 cycle (Supplementary material S3).

### 3.4 Associations between the serum 25(OH)D concentration and mortality

**Figure 1:**
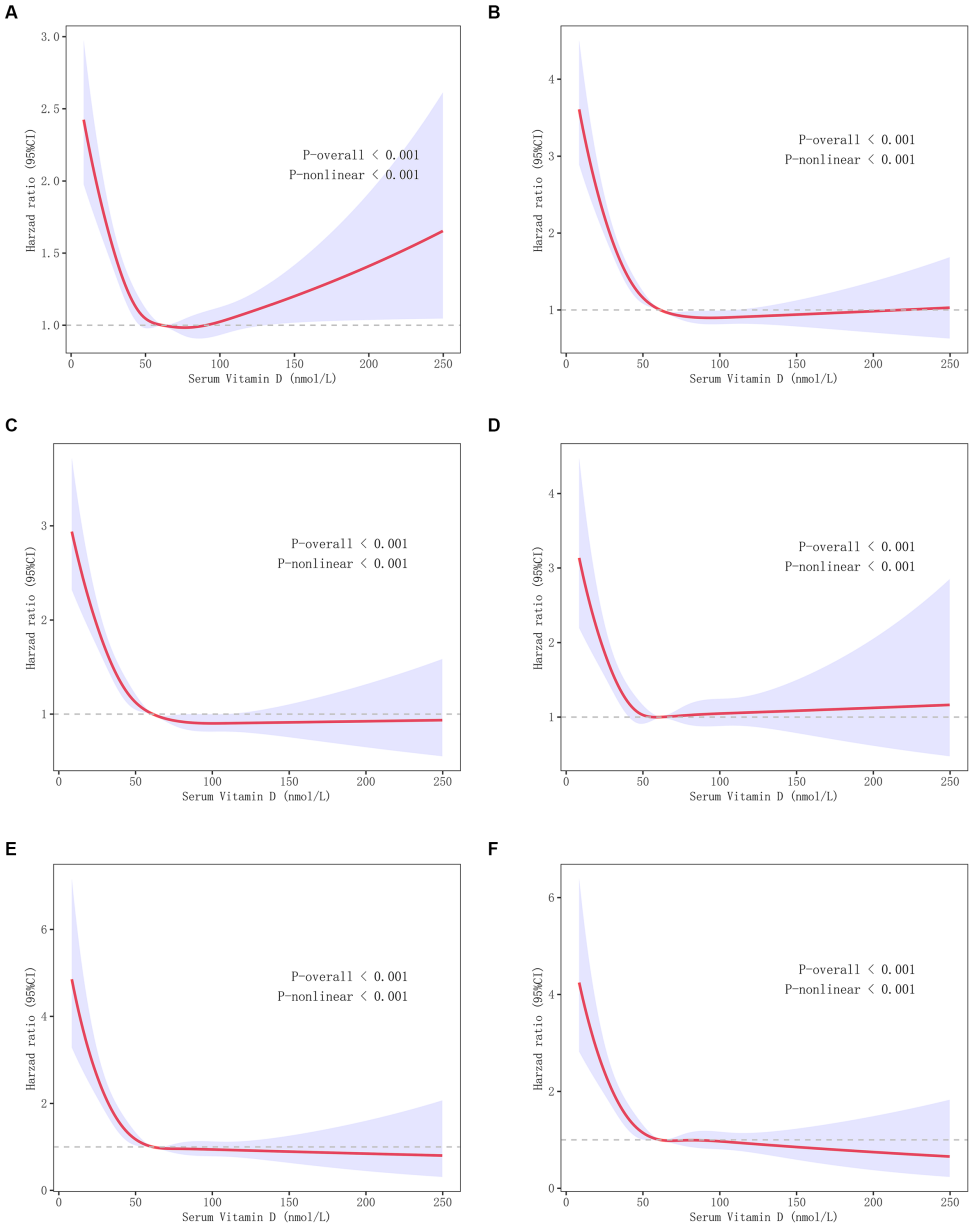
Correlations between the serum 25 (OH) D concentration and CVD and all-cause mortality. **(A)** V-shaped nonlinear correlation between serum 25(OH)D concentration and all-cause mortality in Model 1. **(B)** The L-relationship between serum 25 (OH) D concentration and all-cause mortality in Model 2. **(C)** The L-shaped relationship between the serum 25 (OH) D concentration and all-cause mortality in Model 3. **(D–F)** The L-shaped relationship between the serum 25 (OH) D concentration and CVD mortality in models 1 **(D)**, 2 **(E)**, and 3 **(F)**. Model 1, without adjusting covariates; Model 2, adjusting for covariates such as age, education level, PIR, sex, and race; Model 3, adjusted for include Model 2 covariates and disease history, as well as covariates such as smoking and alcohol consumption.

After stratification by serum 25(OH)D deficiency status, the baseline characteristics of the study population are shown in Supplementary material S4. Among the 426,438 individuals, 6,870 had all-cause mortality. There was a V-shaped nonlinear correlation between the serum 25(OH)D concentration and all-cause mortality in Model 1 (*p* < 0.001 for nonlinearity; Figure 1A) and an L-shaped nonlinear association in Models 2 and 3 (*p* < 0.001 for nonlinearity; Figures 1B,C). Moreover, we studied the relationship between serum 25(OH)D levels and CVD mortality and found 2,120 CVD deaths. According to the three models established, it was found that the serum vitamin D concentration was associated with CVD mortality in a nonlinear L-shaped fashion (*p* < 0.001 for nonlinear; Figures 1D–F).

### 3.5 Sensitivity analysis

The sensitivity analysis was partitioned into five parts: (1) we used the 2000 US Census data for age standardization and observed no significant changes in the serum vitamin D concentration, the incidence rate of vitamin D deficiency, or the trend of dietary vitamin D intake (Supplementary materials S5–S8); (2) after excluding participants with a follow-up time of 2 years, the results showed an L-shaped relationship between the serum 25(OH)D concentration and CVD mortality as well as all-cause mortality (*p* < 0.001 for nonlinearity; Supplementary materials S9, S10). (3) We divided the concentration of serum 25(OH)D into four concentration gradients: (1) < 25 nmol/L, (2) 25.0–49.9 mmoL/L, (3) 50–74.9 nmol/L, and (4) ≥ 75 nmol/L. We adjusted for multiple variables using 25.0–49.9 nmol/L subgroup as the reference standard for all-cause mortality. The results showed that the hazard ratio (HR) and 95% confidence interval (CI) were 1.48 (1.18–1.86) for the <25 nmol/L group, 0.74 (0.68–0.81) for the 50–74.9 nmol/L group and 0.66 (0.60–0.73) for the ≥75 nmol/L group (Supplementary material S11). (5) CVD mortality was greater when the concentration was lower than 25 nmol/L. The other subgroups of HR and 95% CI subgroups were as follows: 1.76 (1.22–2.53) for the <25 nmol/L group; 0.68 (0.58–0.80) for the 50–74.9 nmol/L group; and 0.67 (0.56–0.79) for the ≥75 nmol/L group (Supplementary material S12). We investigated the impact of serum vitamin D levels on all-cause and cardiovascular mortality in both the male and female groups. We observed that, in each sex subgroup, there was an L-shaped nonlinear relationship between 25(OH)D concentration and mortality risk. These findings are largely consistent with previous conclusions (Supplementary material S13).

## 4 Discussion

Our analysis revealed an upwards trend in the serum 25(OH)D concentrations among American adults from 2001 to 2018. Furthermore, we identified an L-shaped correlation between serum 25(OH)D levels and both CVD and all-cause mortality, which became more pronounced when the serum 25(OH)D concentration decreased to less than 50 nmol/L. By eliminating reverse causality, converting the concentration of 25-hydroxyvitamin D [25(OH)D] into categorical variables, and conducting sensitivity analysis using methods such as stratification by gender, we have confirmed the reliability of these results. Our conclusions were corroborated through an exhaustive stratification and sensitivity analysis.

Cui et al. reported on the trends in vitamin D levels during the 2001–2018 NHANES survey cycle, focusing on the changes in the proportions of patients with different vitamin D concentrations. Their overall conclusion aligns with our study, which showed a general decrease in the proportion of the population with a serum vitamin D concentrations < 50 nmol/L (7). This consistent trend may be attributed to increased public health awareness about the role of vitamin D, improvements in living standers, and a growing understanding of the negative health consequences associated with vitamin D deficiency. Such awareness likely spurred interventions, including greater emphasis on dietary vitamin D intake and more widespread use of supplements, Supporting this, studies have shown the beneficial effects of sun exposure in ameliorating vitamin D deficiency (18, 19). Nevertheless, the need for additional vitamin D supplementation remains critical, especially for those at high risk of deficiency (20). However, our study, using finer stratification, draws additional conclusions on temporal trends. Our study highlights an escalating trend in the serum 25(OH)D concentration among American adults, particularly in elderly people (≥ 60 years old) and female, which parallels reports from the NHANES study for the period 1988–2010 (6). Previous studies overlooked income and education-based stratification, factors that can significantly affect serum 25(OH)D levels. While no significant vitamin D deficiency fluctuations were observed in our study population, disaggregation by age, sex, or income revealed stark disparities. Serum 25(OH)D levels among 20–39-year-olds remained stable during the 1988–2010 survey phase (6). However, this age bracket exhibited the highest vitamin D deficiency rates in a 2001–2018 study, despite the absence of an age-stratified trend analysis (7). Our study reveals a decline in serum vitamin D and a rise in deficiency among this demographic, contrasting with decreased deficiency in individuals aged 40 and older. Changes in lifestyle patterns may explain this discrepancy, as indicated by increases in indoor occupations, decreases in outdoor activities, and shifts in dietary intake in younger cohorts (21–23). Therefore, a decrease in dietary intake or vitamin D supplementation alone cannot fully explain the observed serum 25(OH)D concentration and the simultaneous increase in vitamin D deficiency in the 20–39 age cohort. Obesity and lack of exercise were noted as primary vitamin D causes of deficiency causes in young populations in an Australian study (21).

In addition, we discerned a significant increase in 25(OH)D levels in more educated and higher-income (PIR ≥ 4) individuals. Individuals lacking college education revealed no significant increase in the serum vitamin D concentration, with deficiency rates consistently exceeding 30%. Consequently, we posit that these specific populations warrant special attention. Interestingly, a downwards trend in vitamin D deficiency incidence prevalence was detected in high-income groups (PIR ≥ 4), suggesting a relationship between 25(OH)D levels and economic income. In the low-income groups (PIR ≤ 1), no significant shifts in serum 25(OH)D concentration were observed across the nine survey cycles. However, a persistently high prevalence of vitamin D deficiency, approximately 30%, was observed in low-income populations, aligning with findings from other demographic studies. For instance, studies involving UK children have indicated that low household income as a significant risk factor for vitamin D deficiency (23). Similarly, a separate study of Chinese women of reproductive age identified a clear correlation between economic income level and vitamin D deficiency incidence (24). A systematic review echoed these findings, analyzing populations in 29 low- and middle-income countries (25). A density-equalizing mapping analysis revealed that, globally, epidemiological surveys of vitamin D are extremely limited, especially in Asian, African, and South American countries (26). The prevalence rates of vitamin D deficiency that are affected by poverty, worldwide, and the extent to which comprehensive epidemiological studies are conducted is influenced by poverty. This indicates that it is very necessary to conduct similar surveys in less developed countries and regions.

To further elucidate the connection between vitamin D and overall mortality, we adjusted for an array of covariates, such as sociodemographic factors and health conditions. Following this adjustment, an L-shaped inverse association was observed between serum 25(OH)D levels and CVD mortality and between 25(OH)D levels and all-cause mortality. Below a certain inflection point of vitamin D concentration, no additional reduction in CVD or all-cause mortality was observed. Specifically, a serum 25(OH)D concentrations less than 50 nmol/L was significantly negatively correlated with all-cause mortality. With 25(OH)D levels between 50 and 75 nmol/L, all-cause mortality seemed to plateau. Echoing the findings of previous, we found a nonlinear association between 25(OH)D levels and both CVD and all-cause mortality. Specifically, when serum vitamin D concentrations dipped are less than 25 ng/L, the risk of CVD and all-cause mortality significantly increased (27–30).

Based on our results and previous studies, boosting vitamin D intake could benefit public health and potentially lower CVD and all-cause mortality. Given the L-shaped relationship between the serum vitamin D concentration and all-cause mortality, vitamin D supplementation may primarily benefit deficient individuals, emphasizing the need for wide-scale screening and intervention within these groups. A recent meta-analysis and a Cochrane review indicated that while vitamin D and calcium supplementation may not reduce all-cause mortality, they do decrease the risk of cancer death (8). This is further supported by the RCTs, which revealed a significant reduction in cancer mortality (9, 13). Consistent with our hypothesis, in a real-world study based on the UK Biobank, the prescription of vitamin D supplements did indeed reduce cancer mortality, all-cause mortality, or the risk of respiratory infections (2).

## 5 Strengths and limitations

The current research has the following strengths. First, it offers a comprehensive long-term analysis reflecting serum vitamin D trends and their association with CVD and all-cause mortality across a large population. Second, by employing stratification based on age and socioeconomic factors, this study effectively elucidated vitamin D variations and their impact on deficiency prevalence. Third, after we adjusted for several demographic factors, a detailed exploration of the relationship between the serum 25(OH)D concentration and mortality was performed to further guarantee the accuracy of the study. However, our study has several limitations: (1) the trend-based nature of the study restricts causal interpretation of the observed outcomes; (2) potential biases may emerge from a single measurement of serum vitamin D concentrations; (3) vitamin D deficiency may be influenced by various uncontrollable factors, such as sunlight exposure and genetic variations; (4) recall bias due to self-reported dietary data and disease status; and (5) measurement errors from different serum 25(OH)D assessment methods in the NHANES study could also increase potential error; (6) In the estimation of the association between 25(OH)D concentrations and mortality, the interval with concentrations >125 nmol/L exhibited a wide 95% CI for the HR, indicating a lack of precision in this value interval.

## 6 Conclusion

From 2001 to 2018, serum vitamin D levels in U.S. adults increased, but deficiency rates remained unchanged. We observed an L-shaped correlation between vitamin D levels and all-cause mortality, showing a significant inverse relationship when vitamin D levels are below 50 nmol/L. This highlights the importance of a nationwide survey on serum vitamin D levels to inform targeted public health strategies, particularly for high-risk populations.

## Data availability statement

Publicly available datasets were analyzed in this study. This data can be found at: all data for this study can be obtained from NHANES (https://www.cdc.gov/nchs/nhanes/index.htm).

## Ethics statement

The NHANES study protocol was approved by the Research Ethics Review Board of the National Center for Health Statistics (NCHS) of the Centers for Disease Control and Prevention. The studies were conducted in accordance with the local legislation and institutional requirements. Written informed consent for participation in this study was provided by the participants’ legal guardians/next of kin.

## Author contributions

CH: Data curation, Formal analysis, Funding acquisition, Investigation, Methodology, Software, Visualization, Writing – original draft. MY: Conceptualization, Methodology, Supervision, Writing – review & editing.

## Footnotes

^2^https://wwwn.cdc.gov/nchs/nhanes/tutorials/Weighting.aspx
