# Supplementary figures and images for: Trends of serum 25(OH) vitamin D and association with cardiovascular disease and all-cause mortality: from NHANES survey cycles 2001–2018
Source: Front Nutr. 2024 Feb 2;11:1328136. doi: 10.3389/fnut.2024.1328136 (PMC10869563; doi:10.3389/fnut.2024.1328136)

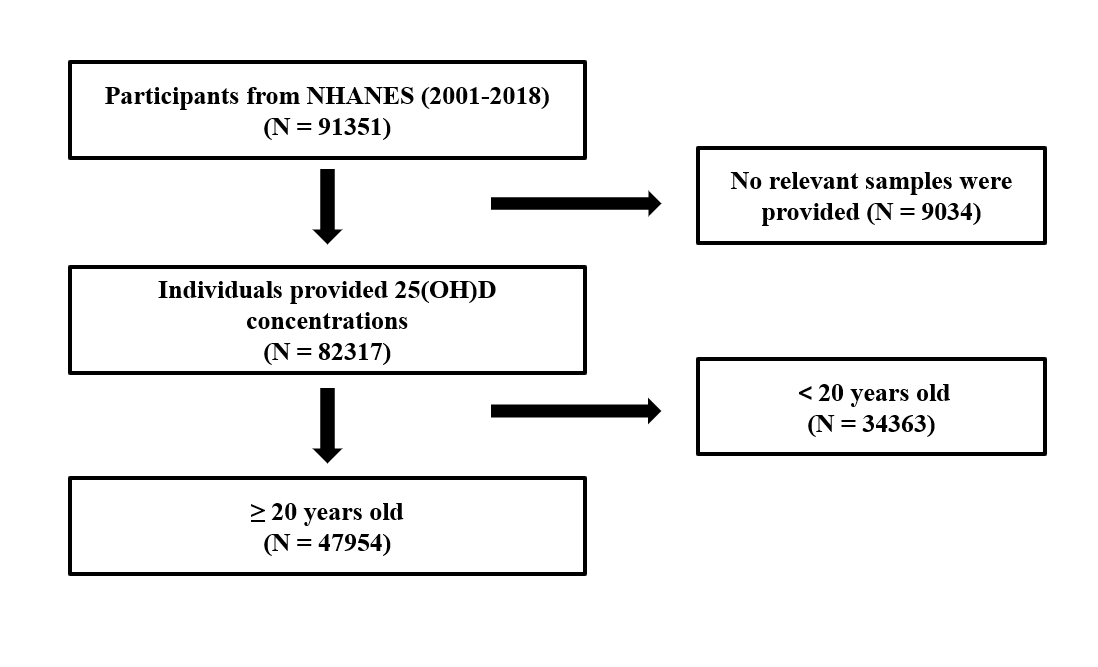


**Supplementary 1. Flowchart to demonstrate selection process.**

Supplement: Supplementary file 1 [file Table_1.docx]
