# Supplementary material for: Trends of serum 25(OH) vitamin D and association with cardiovascular disease and all-cause mortality: from NHANES survey cycles 2001–2018
Source: Front Nutr. 2024 Feb 2;11:1328136. doi: 10.3389/fnut.2024.1328136 (PMC10869563; doi:10.3389/fnut.2024.1328136)
Supplement: Supplementary file 2 [file Table_2.docx]

**Supplementary 2. Trends of dietary supplements vitamin D intake for US Adults by NHANES Survey Cycle, 2007 to 2018**

| Trends of Vitamin D intake from dietary supplements (mcg), weighted mean (95%CI) ^a^ | | | | | | | Difference, 2017-2018 vs 2007-2008 (95% CI) ^b^ | P Value for Trend |
| --- | --- | --- | --- | --- | --- | --- | --- | --- |
|  | 2007-2008  (n=5420) | 2009-2010  (n=5762) | 2011-2012  (n=4801) | 2013-2014  (n=5588) | 2015-2016  (n=5474) | 2017-2018  (n=5265) |  |  |
| **Overall ^c^** | 17.4 (13.3-21.6) | 26.3 (21.9-30.7) | 34.8 (29.8-39.9) | 36.0 (32.2-39.8) | 59.2 (47.9-70.5) | 54.0 (46.5-61.4) | 36.5 (27.6-46.4) | ＜0.001 |
| **Age** |  |  |  |  |  |  |  |  |
| 20-39 | 11.4 (10.4-12.4) | 17.0 (13.3-20.7) | 22.5 (18.6-26.5) | 31.2 (24.5-37.8) | 51.7 (33.0-70.3) | 34.4 (27.0-41.8) | 23.0 (15.2-30.7) | ＜0.001 |
| 40-59 | 18.6 (14.7-22.5) | 32.1 (23.6-40.6) | 41.8 (32.4-51.3) | 41.7 (36.9-46.4) | 61.8 (44.3-79.4) | 61.9 (53.8-70.0) | 34.1 (12.2-55.9) | ＜0.001 |
| ≥60 | 18.6 (14.7-22.5) | 32.1 (23.6-40.6) | 41.8 (32.4-51.3) | 41.7 (36.9-46.4) | 61.8 (44.3-79.4) | 61.9 (53.8-70.0) | 43.3 (34.0-52.6) | ＜0.001 |
| **Sex** |  |  |  |  |  |  |  |  |
| Men | 17.0 (8.2-25.8) | 25.6 (18.0-33.2) | 30.4 (21.9-38.9) | 29.3 (25.3-33.3) | 42.4 (37.1-47.6) | 53.9 (41.0-66.9) | 36.9 (20.6-53.3) | ＜0.001 |
| Women | 17.7 (15.2-20.2) | 26.8 (21.8-31.7) | 38.0 (31.3-44.7) | 40.6 (35.1-46.2) | 71.0 (54.7-87.3) | 54.0 (45.5-62.4) | 36.3 (27.1-45.4) | ＜0.001 |
| **Income-to-poverty ratio ^d^** |  |  |  |  |  |  |  |  |
| ≤1 | 12.6 (11.0-14.2) | 40.4 (3.3-77.6) | 40.7 (13.0-68.4) | 23.6 (19.4-27.8) | 87.9 (38.8-136.9) | 45.6 (31.3-60.0) | 33.0 (18.0-48.1) | ＜0.001 |
| 1-3.9 | 15.9 (12.6-19.2) | 22.4 (19.8-25.0) | 28.2 (24.2-32.2) | 38.6 (31.7-45.5) | 49.9 (37.9-62.0) | 48.8 (37.7-60.0) | 33.0 (20.9-45.1) | ＜0.001 |
| 4 | 20.6 (11.3-29.9) | 24.9 (18.9-30.9) | 36.8 (27.9-45.7) | 33.0 (30.3-35.6) | 60.9 (43.2-78.7) | 62.1 (47.8-76.5) | 41.6 (23.7-59.4) | <0.001 |
| **Education level ^e^** |  |  |  |  |  |  |  |  |
| Less than high school | 17.8 (8.66-26.9) | 44.1 (15.2-73.0) | 20.8 (16.8-24.9) | 44.8 (21.5-68.2) | 44.3 (31.9-56.8) | 52.3 (36.1-68.4) | 34.5 (15.1-53.8) | <0.001 |
| High school or equivalent | 15.2 (13.8-16.6) | 22.9 (19.8-26.1) | 42.6 (21.2-64.0) | 36.0 (27.7-44.2) | 47.8 (35.4-60.2) | 52.3 (38.7-65.9) | 37.1 (22.8-51.3) | <0.001 |
| college or more | 18.2 (12.0-24.4) | 23.7 (20.5-27.0) | 35.3 (28.9-41.7) | 34.7 (31.3-38.2) | 64.0 (46.5-81.5) | 54.8 (45.1-64.4) | 36.6 (24.6-48.5) | <0.001 |
| **Race** |  |  |  |  |  |  |  |  |
| Mexican American ^f^ | 11.0 (10.1-11.8) | 15.3 (12.6-18.1) | 19.4 (13.9-24.9) | 30.0 (16.4-43.5) | 45.3 (24.4-66.2) | 35.9 (31.6-40.3) | 25.0 (20.2-29.8) | <0.001 |
| Non-Hispanic White ^g^ | 18.5 (13.5-23.5) | 27.9 (22.5-33.4) | 36.8 (30.9-42.8) | 34.5 (31.1-38.0) | 65.9 (49.9-82.0) | 57.4 (48.3-66.6) | 38.9 (28.1-49.8) | <0.001 |
| Non-Hispanic Black ^h^ | 12.3 (11.0-13.6) | 23.9 (14.4-22.5) | 32.1 (20.6-43.6) | 43.4 (20.8-65.9) | 44.2 (38.5-50.0) | 60.6 (30.0-91.2) | 48.3 (16.1-80.5) | <0.001 |
| Other ^i^ | 13.6 (11.1-016.0) | 18.9 (13.7-24.0) | 28.0 (22.2-33.9) | 43.5 (18.7-68.3) | 35.1 (31.4-38.8) | 41.9 (36.9-46.9) | 28.3 (22.5-34.2) | <0.001 |

a Data were adjusted for NHANES survey weights to be nationally representative.

b Values may not equal the difference between the beginning and ending estimates because of rounding.

c Dietary supplement vitamin D intake had missing values in every survey cycle. 1398, 1616, 1380, 1470, 1419, 1422 samples reported dietary supplement vitamin D intake in 2007-2008, 2009-2010, 2011-2012, 2013-2014, 2015-2016, 2017-2018 survey cycles respectively.

d 3078 (9.5%) samples did not report PIR missing values.

e 40 (0.1%) samples did not report education level missing values.

f Stratification by ethnic characteristics leaded to stratum with a single PSU. Samples in the stratum with the singleton PSU were removed. 95 (2%) samples removed.

g 32 (0.2%) samples removed.

h 112 (1.6%) samples removed.

i 3 (0.04%) samples removed.
