# Supplementary material for: Trends of serum 25(OH) vitamin D and association with cardiovascular disease and all-cause mortality: from NHANES survey cycles 2001–2018
Source: Front Nutr. 2024 Feb 2;11:1328136. doi: 10.3389/fnut.2024.1328136 (PMC10869563; doi:10.3389/fnut.2024.1328136)
Supplement: Supplementary file 3 [file Table_3.docx]

**Supplementary 3. Trends of food vitamin D intake for US Adults by NHANES Survey Cycle, 2007 to 2018**

| Trends of Vitamin D intake from dietary food (mcg), weighted mean (95%CI) ^a^ | | | | | | | Difference, 2017-2018 vs 2007-2008 (95% CI) ^b^ | P Value for Trend |
| --- | --- | --- | --- | --- | --- | --- | --- | --- |
|  | 2007-2008  (n=5420) | 2009-2010  (n=5762) | 2011-2012  (n=4801) | 2013-2014  (n=5047) | 2015-2016  (n=5017) | 2017-2018  (n=4742) |  |  |
| **Overall** | 4.37 (4.12-4.62) | 5.14 (4.84-5.43) | 4.69 (4.40-4.99) | 4.75 (4.43-5.07) | 4.70 (4.39-5.01) | 4.24 (3.97-4.51) | -0.13 (-0.52-0.26) | ＜0.001 |
| **Age** |  |  |  |  |  |  |  |  |
| 20-39 | 4.19 (3.81-4.56) | 5.05 (4.69-5.41) | 4.57 (4.16-4.98) | 4.65 (4.21-5.09) | 4.75 (4.04-5.46) | 4.09 (3.78-4.41) | -0.1 (-0.60-0.41) | 0.002 |
| 40-59 | 4.66 (4.26-5.05) | 5.19 (4.57-5.81) | 4.75 (4.27-5.22) | 4.79 (4.25-5.33) | 4.73 (4.38-5.07) | 4.04 (3.62-4.47) | -0.61 (-1.21--0.01) | 0.044 |
| ≥60 | 4.18 (4.01-4.35) | 5.18 (4.91-5.45) | 4.78 (4.48-5.09) | 4.83 (4.40-5.27) | 4.59 (4.24-4.94) | 4.67 (3.93-5.40) | 0.49 (-0.29-1.28) | ＜0.001 |
| **Sex** |  |  |  |  |  |  |  |  |
| Men | 5.01 (4.57-5.45) | 5.88 (5.48-6.28) | 5.50 (5.07-5.93) | 5.52 (4.92-6.13) | 5.13 (4.69-5.57) | 4.80 (4.47-5.13) | -0.21 (-0.78-0.36) | 0.001 |
| Women | 3.80 (3,55-4.06) | 4.45 (4.15-4.75) | 3.92 (3.67-4.16) | 4.02 (3.82-4.22) | 4.30 (3.99-4.62) | 3.72 (3.34-4.11) | -0.08 (-0.56-0.41) | 0.006 |
| **Income-to-poverty ratio ^c^** |  |  |  |  |  |  |  |  |
| ≤1 | 3.81 (3.42-4.20) | 4.94 (4.53-5.35) | 4.73 (3.90-5.57) | 4.54 (4.22-4.87) | 4.26 (3,82-4.70) | 4.08 (3.69-4.48) | 0.27 (-0.31-0.85) | 0.002 |
| 1-3.9 | 4.31 (3.95-4.67) | 4.89 (4.56-5.21) | 4.53 (4.25-4.82) | 4.61 (4.18-5.05) | 4.65 (4.28-5.03) | 4.24 (4.01-4.46) | -0.07 (-0.51-0.37) | 0.027 |
| ≥4 | 4.67 (4.38-4.96) | 5.55 (4.94-6.15) | 4.89 (4.42-5.35) | 5.05 (4.52-5.57) | 4.93 (4.43-5.44) | 4.31 (3.78-4.84) | -0.36 (-0.99-0.27) | 0.048 |
| **Education level ^d^** |  |  |  |  |  |  |  |  |
| Less than high school | 4.00 (3.60-4.41) | 4.38 (4.13-4.64) | 4.74 (4.08-5.40) | 4.42 (3.99-4.85) | 4.63 (4.03-5.23) | 4.18 (3.72-4.63) | 0.17 (-0.46-0.80) | 0.339 |
| High school or equivalent | 3.90 (3.62-4.19) | 5.17 (4.64-5.70) | 4.57 (4.18-4.97) | 4.29 (3.80-4.78) | 4.54 (3.89-5.19) | 4.32 (3.91-4.73) | 0.42 (-0.11-0.94) | 0.001 |
| college or more | 4.72 (4.42-5.02) | 5.35 (4.93-5.77) | 4.72 (4.36-5.07) | 4.99 (4.50-5.47) | 4.76 (4.39-5.14) | 4.22 (3.88-4.55) | -0.5 (-0.97--0.35) | 0.002 |
| **Race** |  |  |  |  |  |  |  |  |
| Mexican American ^e^ | 4.54 (3.95-5.12) | 5.02 (4.49-5.56) | 4.64 (4.15-5.12) | 4.33 (3.85-4.82) | 4.70 (4.15-5.24) | 4.39 (3.84-4.94) | -0.15 (-0.10-0.70) | 0.508 |
| Non-Hispanic White ^f^ | 4.50 (4.18-4.82) | 5.39 (4.93-5.85) | 4.86 (4.47-5.26) | 5.00 (4.57-5.43) | 4.87 (4.39-5.35) | 4.25 (3.87-4.64) | -0.25 (-0.77-0.27) | 0.003 |
| Non-Hispanic Black ^g^ | 3.28 (2.90-3.67) | 4.08 (3.74-4.42) | 3.98 (3.71-4.25) | 3.88 (3.65-4.11) | 3.92 (3.63-4.20) | 3.68 (3.29-4.07) | 0.40 (-0.18-0.97) | 0.034 |
| Other ^h^ | 4.31 (3.81-4.81) | 4.69 (4.41-4.97) | 4.46 (4.17-4.75) | 4.52 (4.14-4.90) | 4.51 (4.24-4.79) | 4.52 (4.01-5.02) | 0.21 (-0.54-0.95) | 0.817 |

a Data were adjusted for NHANES survey weights to be nationally representative.

b Values may not equal the difference between the beginning and ending estimates because of rounding.

c 3078 (9.5%) samples reported PIR missing values.

d 40 (0.1%) samples reported education level missing values.

e Stratification by ethnic characteristics leaded to stratum with a single PSU. Samples in the stratum with the singleton PSU were removed. 232 (5.7%) samples removed.

f 31 (0.3%) samples removed.

g 99 (1.7%) samples removed.

h 2 (0.03%) samples removed.
