# Supplementary material for: Trends of serum 25(OH) vitamin D and association with cardiovascular disease and all-cause mortality: from NHANES survey cycles 2001–2018
Source: Front Nutr. 2024 Feb 2;11:1328136. doi: 10.3389/fnut.2024.1328136 (PMC10869563; doi:10.3389/fnut.2024.1328136)
Supplement: Supplementary file 4 [file Table_4.docx]

**Supplementary 4. Baseline Characteristics of Participants according to Serum 25(OH)D deficiency in NHANES from 2001-2018.**

|  | Participants, (weighted %) | |
| --- | --- | --- |
| Characteristics | 25(OH)D＜50nmol/L | 25(OH)D ≥ 50nmol/L |
| Participants, No. ^a^ | 14257.00 | 30204.00 |
| Weighted Participants No. | 451783587.00 | 1399091012.00 |
| Age, year, mean (SE) | 44.11 (0.23) | 48.20 (0.21) |
| Sex (%) |  |  |
| Female | 53.48 | 51.34 |
| Male | 46.52 | 48.66 |
| Income-to-poverty ratio, mean (SE) | 2.52 (0.03) | 3.17 (0.03) |
| Education level (%) |  |  |
| Less than high school | 22.44 | 14.70 |
| High school or equivalent | 24.73 | 23.47 |
| college or more | 52.83 | 61.83 |
| Race (%) |  |  |
| Mexican American | 14.00 | 6.51 |
| Non-Hispanic White | 41.29 | 77.17 |
| Non-Hispanic Black | 27.36 | 53.88 |
| Other | 17.35 | 10.93 |
| Smoking (%) |  |  |
| Never Smoker | 55.86 | 53.53 |
| Former Smoker | 18.62 | 26.84 |
| Current Smoker | 25.53 | 19.64 |
| Alcohol (%) |  |  |
| Non-alcohol use | 71.90 | 79.64 |
| Alcohol use | 28.10 | 20.36 |
| T2DM (%) |  |  |
| Non-T2DM | 89.11 | 91.37 |
| T2DM | 10.89 | 8.63 |
| Hypertension (%) |  |  |
| Non-Hypertension | 68.60 | 68.99 |
| Hypertension | 31.40 | 31.01 |
| Cancer (%) |  |  |
| Non-cancer | 93.76 | 89.27 |
| Cancer | 6.24 | 10.73 |

a 3493 (7.3%) samples reported serum 25(OH)D missing values.
