# Supplementary material for: Trends of serum 25(OH) vitamin D and association with cardiovascular disease and all-cause mortality: from NHANES survey cycles 2001–2018
Source: Front Nutr. 2024 Feb 2;11:1328136. doi: 10.3389/fnut.2024.1328136 (PMC10869563; doi:10.3389/fnut.2024.1328136)
Supplement: Supplementary file 6 [file Table_6.docx]

**Supplementary 6. Age-adjusted trends of vitamin D deficiency among adults aged 20 years or older by NHANES survey cycle, 2001 to 2018.**

| Age-adjusted and weighted percentage (%) of individuals with vitamin D deficiency, % (95% CI) ^a^ | | | | | | | | | P Value for Trend |
| --- | --- | --- | --- | --- | --- | --- | --- | --- | --- |
| 2001-2002 (n=4340) | 2003-2004 (n=4243) | 2005-2006 (n=4292) | 2007-2008 (n=4244) | 2009-2010 (n=5182) | 2011-2012 (n=4556) | 2013-2014 (n=4953) | 2015-2016 (n=4668) | 2017-2018 (n=4335) |  |
| 22.8 (19.8-25.8) | 30.5 (24.5-36.5) | 21.4 (17.8-25.0) | 26.2 (22.5-29.9) | 25.9 (22.1-29.8) | 25.0 (19.9-30.2) | 25.1 (21.3-29.0) | 23.8 (19.3-28.2) | 22.6 (18.9-26.4) | 0.359 |

a Age adjust was constructed by recalculating sample weights based on the National Center for Health Statistics Guidelines for Analysis of Trends. Values may not equal the difference between the beginning and ending estimates because of rounding.
