# Supplementary material for: Trends of serum 25(OH) vitamin D and association with cardiovascular disease and all-cause mortality: from NHANES survey cycles 2001–2018
Source: Front Nutr. 2024 Feb 2;11:1328136. doi: 10.3389/fnut.2024.1328136 (PMC10869563; doi:10.3389/fnut.2024.1328136)
Supplement: Supplementary file 9 [file Table_9.docx]

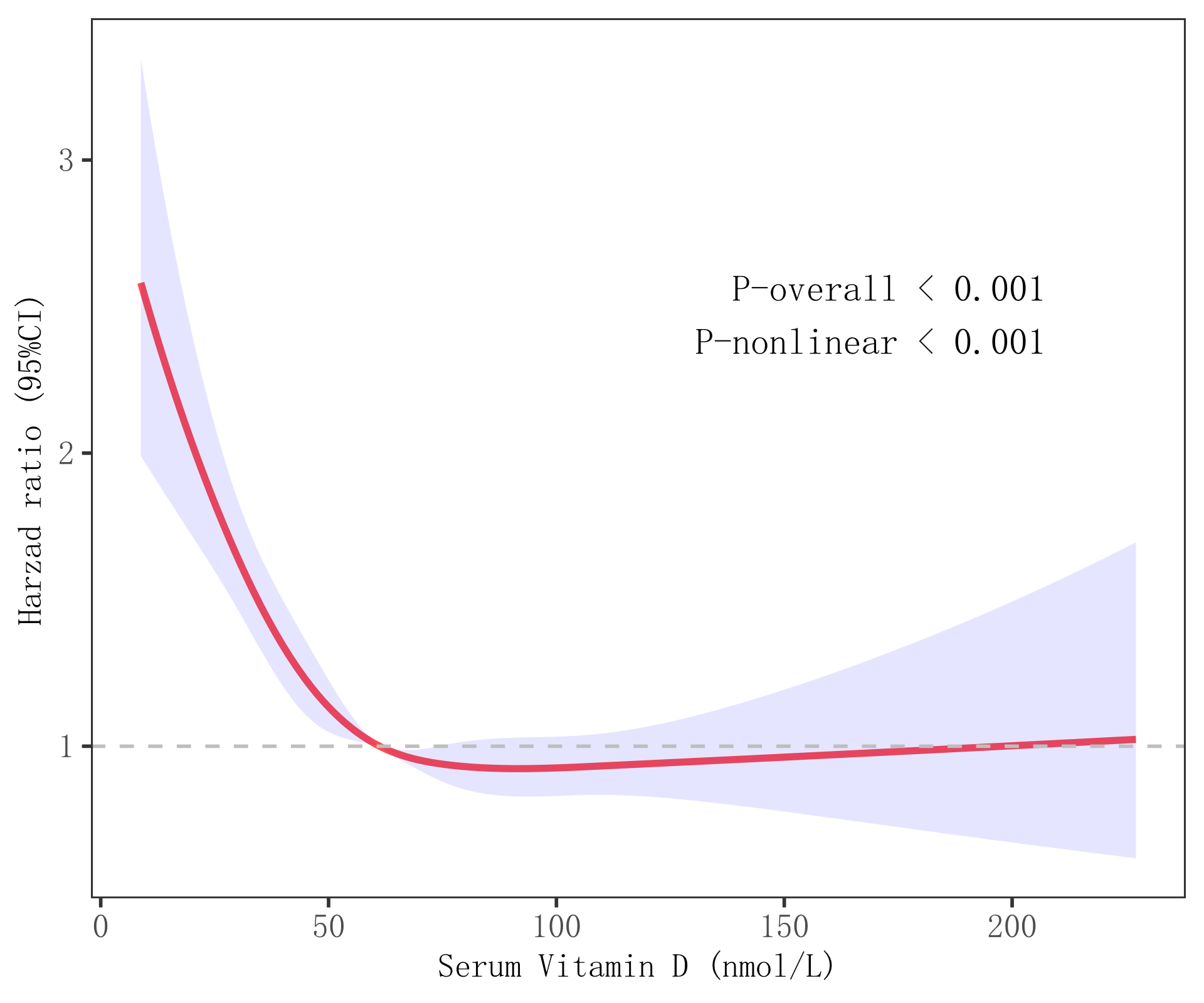


Supplementary 9. The relationship between serum vitamin D and all-cause mortality after excluding participants who were followed up for less than 2 years.
