# Supplementary material for: Trends of serum 25(OH) vitamin D and association with cardiovascular disease and all-cause mortality: from NHANES survey cycles 2001–2018
Source: Front Nutr. 2024 Feb 2;11:1328136. doi: 10.3389/fnut.2024.1328136 (PMC10869563; doi:10.3389/fnut.2024.1328136)
Supplement: Supplementary file 13 [file Table_13.docx]

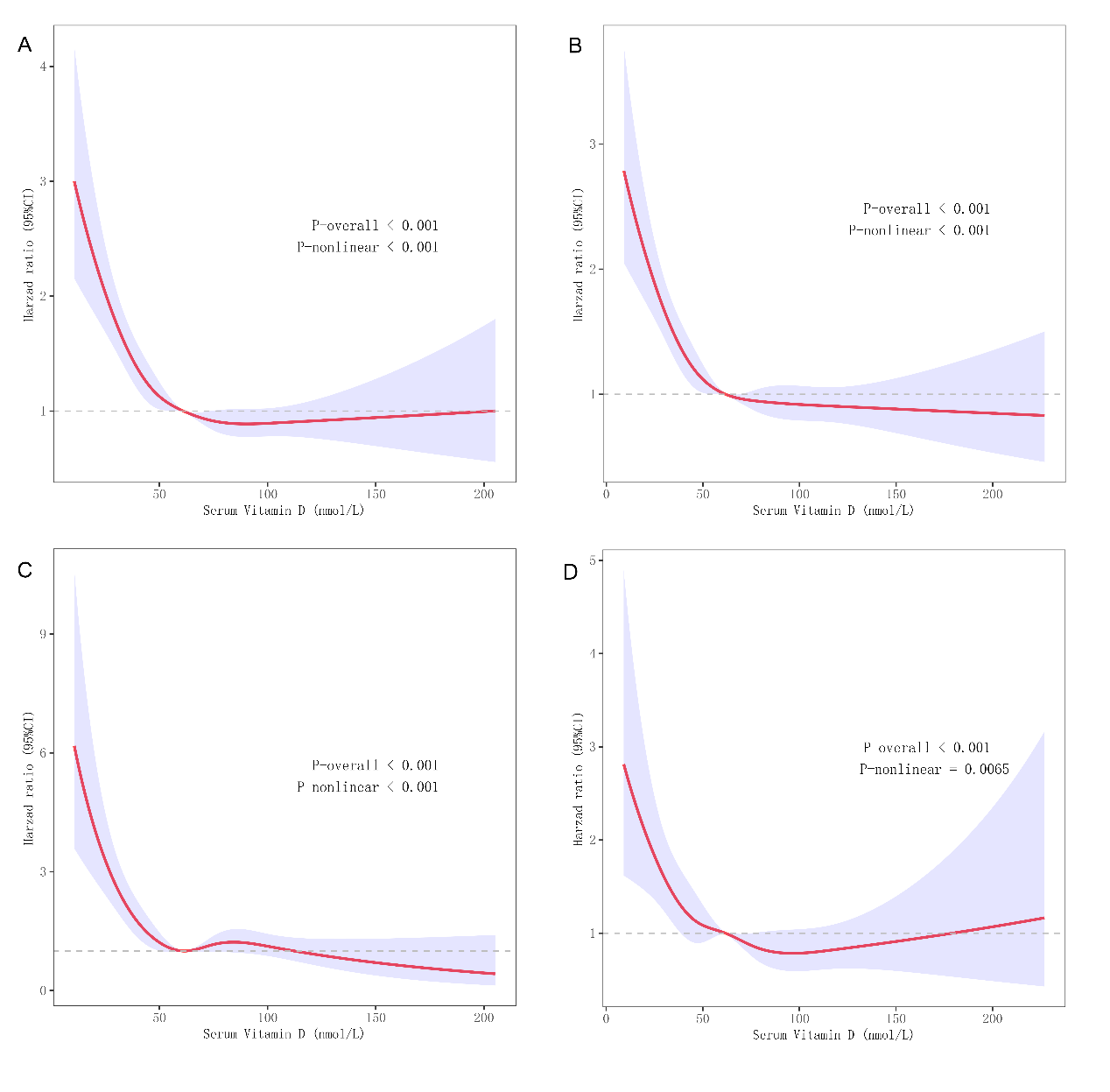


Supplementary 13. The Association between the serum 25(OH)D concertation and All-Cause Mortality or Cardiovascular Mortality in Male and Female Subgroups. A. An L-shaped relationship is observed between the serum 25(OH)D concertation and all-cause mortality in males; B. An L-shaped relationship is noted between the serum 25(OH)D concertation and all-cause mortality in females; C. In males, the serum 25(OH)D concertation demonstrate an L-shaped association with cardiovascular mortality; D. In females, the serum 25(OH)D concertation are associated with cardiovascular mortality in an L-shaped pattern.
