# Supplementary material for: Trends of serum 25(OH) vitamin D and association with cardiovascular disease and all-cause mortality: from NHANES survey cycles 2001–2018
Source: Front Nutr. 2024 Feb 2;11:1328136. doi: 10.3389/fnut.2024.1328136 (PMC10869563; doi:10.3389/fnut.2024.1328136)
Supplement: Supplementary file 14 [file Table_14.docx]

**Supplementary 13. Preferred Reporting Items for Complex Sample Survey Analysis (PRICSSA) checklist.**

| **PRICSSA item** | **Description** | **Answer** |
| --- | --- | --- |
| 1.1 Data collection dates | Describe the survey’s data collection dates (e.g., range) to provide historical context that could affect survey responses and nonresponse. | Results: Population characteristics (Line 157-159) |
| 1.2 Data collection mode(s) | Describe the survey’s data collection mode(s). Data collection mode can affect survey responses (e.g., to sensitive questions), including nonresponse, and a survey’s data collection mode may change over time (e.g., during the COVID-19 pandemic). | Methods: Study Design and Population (Line 75-83) |
| 1.3 Target population | State the target population the survey was designed to represent and describe all weighted estimates with respect to this target population. | Methods: Study Design and Population (Line 86-90; Line124-126) |
| 1.4 Sample design | Describe the survey’s sample design, including information about stratification, cluster sampling, and unequal probabilities of selection. | Methods: Statistical analysis  (Line 121-127) |
| 1.5 Survey response rate(s) | State the survey’s response rate and how it was calculated. | Methods: Statistical analysis  (Line 125-126) |
| 2.1 Missingness rates | Report rates of missingness for variables of interest and models, and describe any methods (if any) for dealing with missing data (e.g., multiple imputation). | Methods: Statistical analysis  (Line 137-139) |
| 2.2 Observation deletion | State whether any observations were deleted from the dataset. If observations were deleted, provide a justification. Note: It is best practice to avoid deleting cases and use available subpopulation analysis commands no matter what variance estimation method is used. | Methods: Statistical analysis  (Line 137-139) |
| 2.3 Sample sizes | Include unweighted sample sizes for all weighted estimates. | Methods: Statistical analysis. Details of unweighted sample sizes remain in each table. (Line 129). |
| 2.4 Confidence intervals/standard errors | Include confidence intervals or standard errors when reporting all estimates to inform the reliability/precision of each estimate. | Methods: Statistical analysis. (Line133-135; 139-142) |
| 2.5 Weighting | State which analyses were weighted and specify which weight variables were used in analysis. | Methods: Statistical analysis  (Line 126-129) |
| 2.6 Variance estimation | Describe the variance estimation method used in the analysis and specify which design variables (e.g., PSU/stratum, replicate weights) were used. | Methods: Statistical analysis  (Line 122-126) |
| 2.7 Subpopulation analysis | Describe the procedures used for conducting subpopulation analyses (e.g., Stata’s “subpop” command, SAS’s “domain” command). | The analysis was done in R, and the code can be requested from the author or, if requested by the journal, provided after acceptance (Line 304-305). |
| 2.8 Suppression rules | State whether or not a suppression rule was followed (e.g., minimum sample size or relative standard error). | NA |
| 2.9 Software and code | Report which statistical software was used, comprehensively describe data management and analysis in the manuscript, and provide all statistical software code. | Methods: Statistical analysis  (Line 153-154) |
| 2.10 Singleton problem (as needed) | Taylor Series Linearization requires at least two PSUs per stratum for variance estimation. Sometimes an analysis is being performed and there is only a single PSU in a stratum. There are several possible fixes to this problem, which should be detailed if the singleton problem is encountered. | Methods: Statistical analysis  (Line 138-139 |
| 2.11 Public/restricted data (as needed) | If applicable, state whether the public use or restricted version of the dataset was analyzed. | NA |
| 2.12 Embedded experiments (as needed) | If applicable, provide information about split sample embedded experiments (e.g., mode of data collection or varying participant incentives) and detail whether experimental factors were accounted for in the analyses. | NA |
